# Proton Beam Therapy for the Treatment of Recurrent Human Papillomavirus-Related Multiphenotypic Sinonasal Carcinoma: A Case Report

**DOI:** 10.7759/cureus.83539

**Published:** 2025-05-05

**Authors:** Ken Matsumoto, Shiro Akahani

**Affiliations:** 1 Otolaryngology - Head and Neck Surgery, Kansai Rosai Hospital, Amagasaki, JPN

**Keywords:** head and neck, hmsc, hpv-related multiphenotypic sinonasal carcinoma, local recurrence, proton beam therapy, sinonasal carcinoma

## Abstract

Human papillomavirus-related multiphenotypic sinonasal carcinoma (HMSC) is a rare sinonasal tract tumor. Although HMSC has a favorable prognosis, patients tend to experience local recurrence after the initial therapy; therefore, careful follow-up is required after surgical dissection. Proton beam therapy is one of the therapeutic modalities used to treat sinonasal cancer. This is the first report on the use of proton beam therapy for definitive irradiation in HMSC. Herein, we report the case of a 30-year-old woman with HMSC. The right nasal tumor was surgically resected by endoscopy, and HMSC was diagnosed using pathology. The diagnosis of HMSC was made. Nine months after surgery, a recurrent invasive tumor was found in the right nasal cavity, and surgical treatment was not feasible. Proton beam therapy was administered to the recurrent lesion, resulting in radiographic complete response maintained for nine months. This case suggests that postoperative recurrence must be noted and that proton beam therapy is an option to treat locally advanced lesions.

## Introduction

The human papillomavirus (HPV) plays an oncogenic role in the development of a subset of head and neck squamous cell cancers [1]. High-risk HPV type 16 is detected in up to 80％ of oropharyngeal carcinomas, and HPV status helps clinicians predict clinical outcomes [2,3]. HPV testing can detect high-risk HPV in 20-30% of sinonasal cancers [4,5]. The clinical significance of HPV infection in sinonasal carcinoma is unclear [6].

HPV-related multiphenotypic sinonasal carcinoma (HMSC) is a unique tumor limited to the sinonasal tract [7]. HMSC shows histological features of both salivary gland and squamous cell carcinomas [8].

Immunohistochemical staining for p16, an HPV surrogate marker, is diffusely positive in HMSC. Most cases of HMSC show high-risk HPV type 33 instead of HPV type 16 in oropharyngeal carcinoma, as revealed by rigorous detection methods such as through DNA or RNA in situ hybridization (ISH) [8]. This clinical picture is slightly more common in women than in men. The mean age at diagnosis is approximately 50 years [8]. Most patients with HMSC present with epistaxis and nasal obstruction [9]. HMSC commonly originates from the nasal cavity, with occasional paranasal sinus involvement [8].

HMSC shows slow growth with a favorable prognosis in the clinical setting [10]. It is primarily treated with surgical dissection and occasionally with adjuvant radiation. However, HMSC is associated with a relatively high rate of local recurrence after initial treatment, and there is no established treatment modality for this rare entity [9,10].

Radiation therapy, instead of surgery, is also indicated as the primary treatment for unresectable sinonasal malignant tumors. Proton beam therapy has a higher biological efficacy than photon therapy, achieving not only preservation of at-risk organs but also greater therapeutic benefits in terms of tumor control and survival rates [11].

Here, we report the case of a patient diagnosed with HMSC with confirmed local recurrence after endoscopic surgery. Proton beam therapy targeting the recurrent lesion achieved durable local disease control during follow-up. This report aims to highlight the potential role of proton beam therapy in managing locally recurrent HMSC, a rare and understudied clinical entity.

## Case presentation

**Figure 1:**
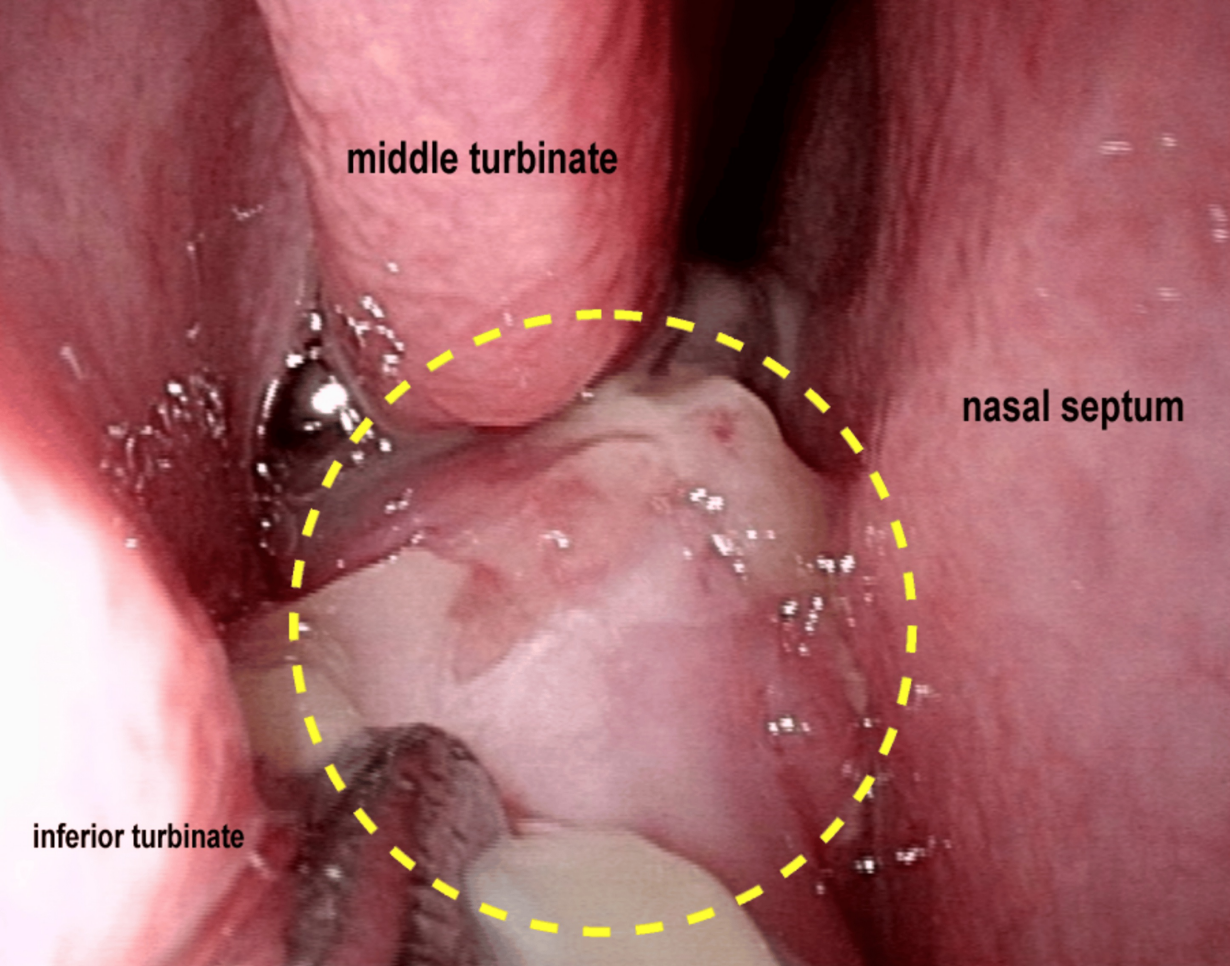
Nasal endoscopy in the right nasal cavity at initial examination Nasal endoscopy showing the hemorrhagic, friable, and necrotic nasal tumor in the right nasal cavity (dashed line).

A 30-year-old woman visited our hospital, presenting with right nasal obstruction that had persisted for three months. The patient did not report epistaxis, anosmia, facial pain, or pressure. The patient had no relevant medical history and was a non-smoker. Nasal endoscopy revealed a nasal tumor in the right common nasal meatus. The tumor was pink in color, hemorrhagic, and friable with a few superficial marginal necroses (Figure 1).

**Figure 2:**
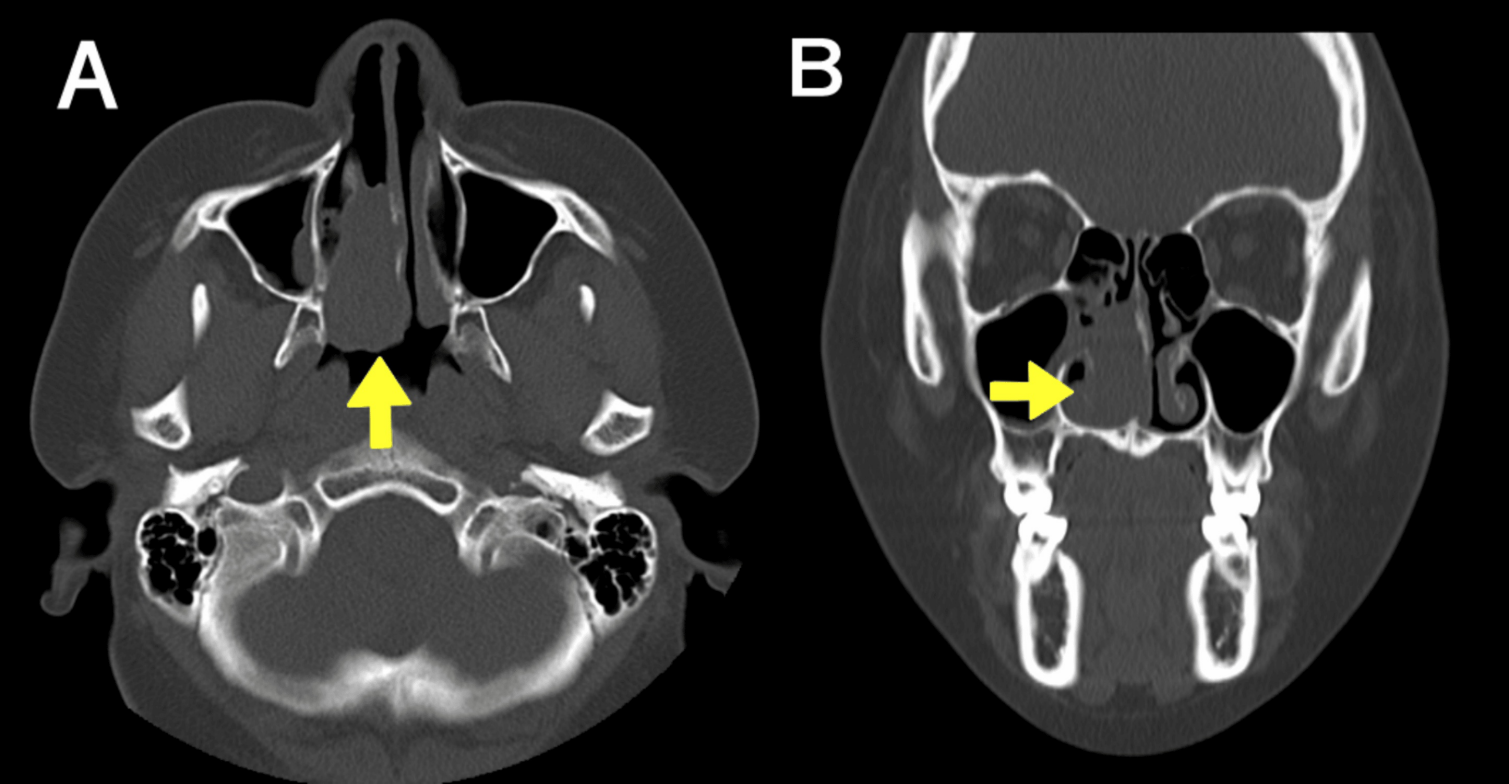
CT imaging before the initial surgical therapy Coronal (A) and axial (B) computed tomography (CT) images of the tumor before endoscopic surgery. The tumor was confined to the right nasal cavity with no bone invasion (arrow).

Computed tomography revealed that the tumor was limited to the right inferior common nasal cavity, and no bone erosion was observed. The tumor was a soft tissue mass of size 12 × 28 × 36 mm^3^ (Figure 2A, 2B).

Endoscopic surgical resection was performed for diagnosis and treatment. The tumor was located in the right nasal septum mucosa and was excised along with the septal cartilage and bone. Histological examination of the dissected specimen revealed a malignant carcinoma.

**Figure 3:**
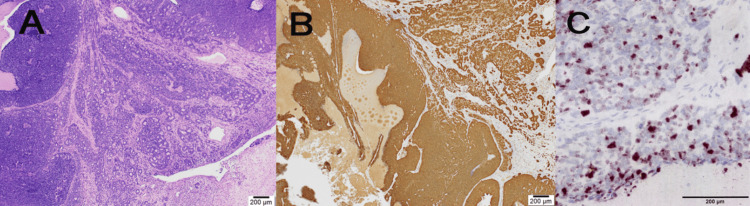
Histopathological and immunohistochemical staining, in situ hybridization of the tumor A. Various forms of tumor cells with basaloid cells, including solid sheets, cribriform nests, and a few ductal structures were observed. Hematoxylin and eosin (H&E) staining (4×). B. Immunohistochemical staining revealed that diffuse tumor cells were positive for p16. C. RNA in situ hybridization indicated that the tumor was positive for high-risk HPV. HPV, human papillomavirus

The tumor cells exhibited high mitotic activity and severe necrosis. Morphologically, the tumor was composed of basaloid cells in various forms, including solid sheets, cribriform nests, and a few ductal structures. The Mindbomb Homolog-1 (MIB‐1) labeling index was 70%. The specimen revealed an infiltrative basaloid carcinoma with salivary gland-like features, consistent with HMSC or adenoid cystic carcinoma (ACC). Immunohistochemistry revealed that the patient was positive for p16 and S-100 proteins. HPV RNA-ISH confirmed the presence of high-risk HPV. The RNA scope™ ISH probe was used for RNA-ISH, and this assay qualitatively detected high-risk HPV types (16, 18, 26, 31, 33, 35, 39, 45, 51, 52, 53, 56, 58, 59, 66, 68, 73, 82) all at once; therefore, we were unable to confirm the HPV types in the tumor tissue. Based on the immunohistochemical history and RNA-ISH, we concluded that the diagnosis was HMSC rather than ACC (Figure 3A, 3B, 3C).

**Figure 4:**
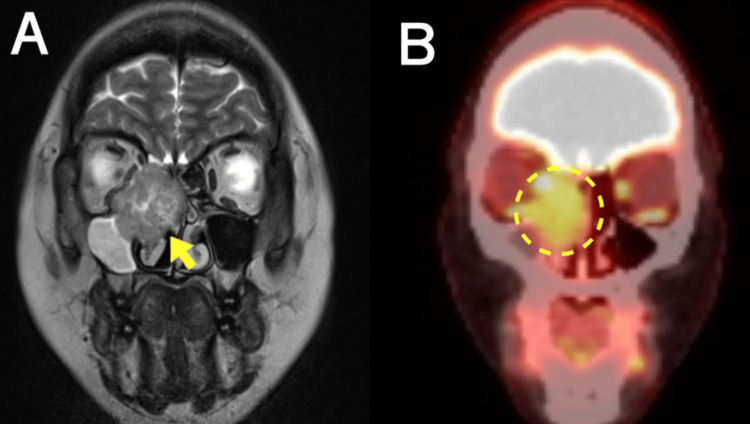
MRI and PET/CT images showing local recurrence after surgery A. Coronal T2 MRI showing an invasive tumor in the orbit and adjacent to the skull base (arrow). B. PET/CT demonstrating elevated FDG uptake in the recurrent tumor (dashed line). MRI, magnetic resonance imaging; PET/CT, positron emission tomography/ computed tomography; FDG, fluorodeoxyglucose

Postoperative positron emission tomography-computed tomography (PET/CT) identified no signs of recurrence or distant metastasis. The follow-up observations were performed without additional treatment. However, the patient was lost to follow-up at our hospital for the next six months after being admitted to another hospital for pregnancy-related complications. Nine months after surgical dissection, the patient presented to our hospital with no symptoms. Nasal endoscopy revealed a round mass in the right middle meatus. Biopsy of the nasal cavity revealed malignant cells, leading to a diagnosis of recurrent HMSC. Magnetic resonance imaging (MRI) revealed a round mass measuring 33 × 37 × 32 mm^3^ in the right nasal cavity as a hyperintensity on T2-weighted images. The tumor had invaded the right orbit and partially contacted the base of the skull. PET/CT confirmed fluorodeoxyglucose (FDG) uptake in the nasal lesion, and the standard uptake value (SUV) of the recurrence lesion was reported to be 6.3; no local or distant metastases were identified (Figure 4A, 4B).

The tumor reappeared in the same nasal cavity, but the recurrent lesion was spherical and had spread around the middle nasal meatus. Although the lesion had infiltrated the orbit with orbital bone invasion, the patient experienced asymptomatic recurrence with no eye movement disorder.

**Figure 5:**
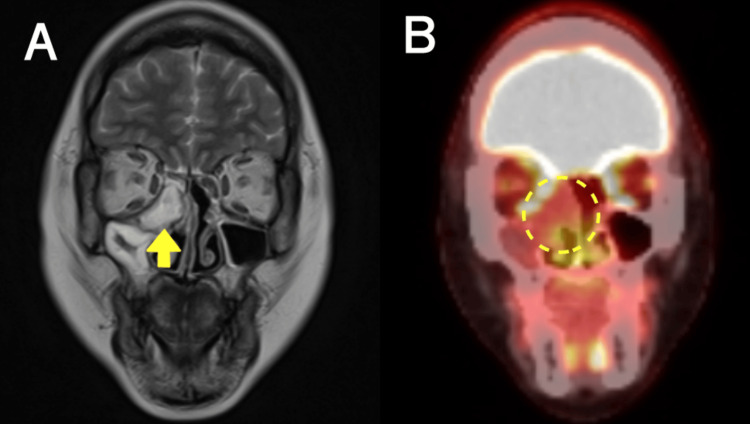
MRI and PET/CT images obtained three months after proton beam therapy A. Coronal T2 MRI showing complete remission with necrotic change (arrow). B. PET/CT demonstrating reduced activity of the mass (dashed line). MRI, magnetic resonance imaging; PET/CT, positron emission tomography/computed tomography

It was difficult to avoid right eye enucleation and maintain an adequate safety margin on the images, and surgical dissection was not feasible. The treatment plan was discussed with various experts, proton beam therapy was recommended, and patient consent was obtained. The patient underwent proton beam therapy targeting the recurrent lesion. A total dose of 70.4 Gy (relative biological effectiveness) was delivered in 32 fractions over six and a half weeks. MRI performed three months after proton therapy revealed that the recurrent lesion was localized, with total internal necrotic changes only in the nasal cavity　(hyperintensity on T2-weighted imaging), and complete remission was achieved. PET/CT demonstrated lower FDG activity (reported SUV of 2.5) in the lesion (Figure 5A, 5B).

The imaging findings demonstrated no changes after proton treatment, and no evidence of recurrence was confirmed after nine months.

According to the National Cancer Institute Common Terminology Criteria for Adverse Events, version 4.0, the patient experienced grade 1 periorbital edema as an acute toxicity and grade 2 nasal congestion as a late toxicity. No acute or late grade ≥3 side effects were observed.

**Figure 6:**
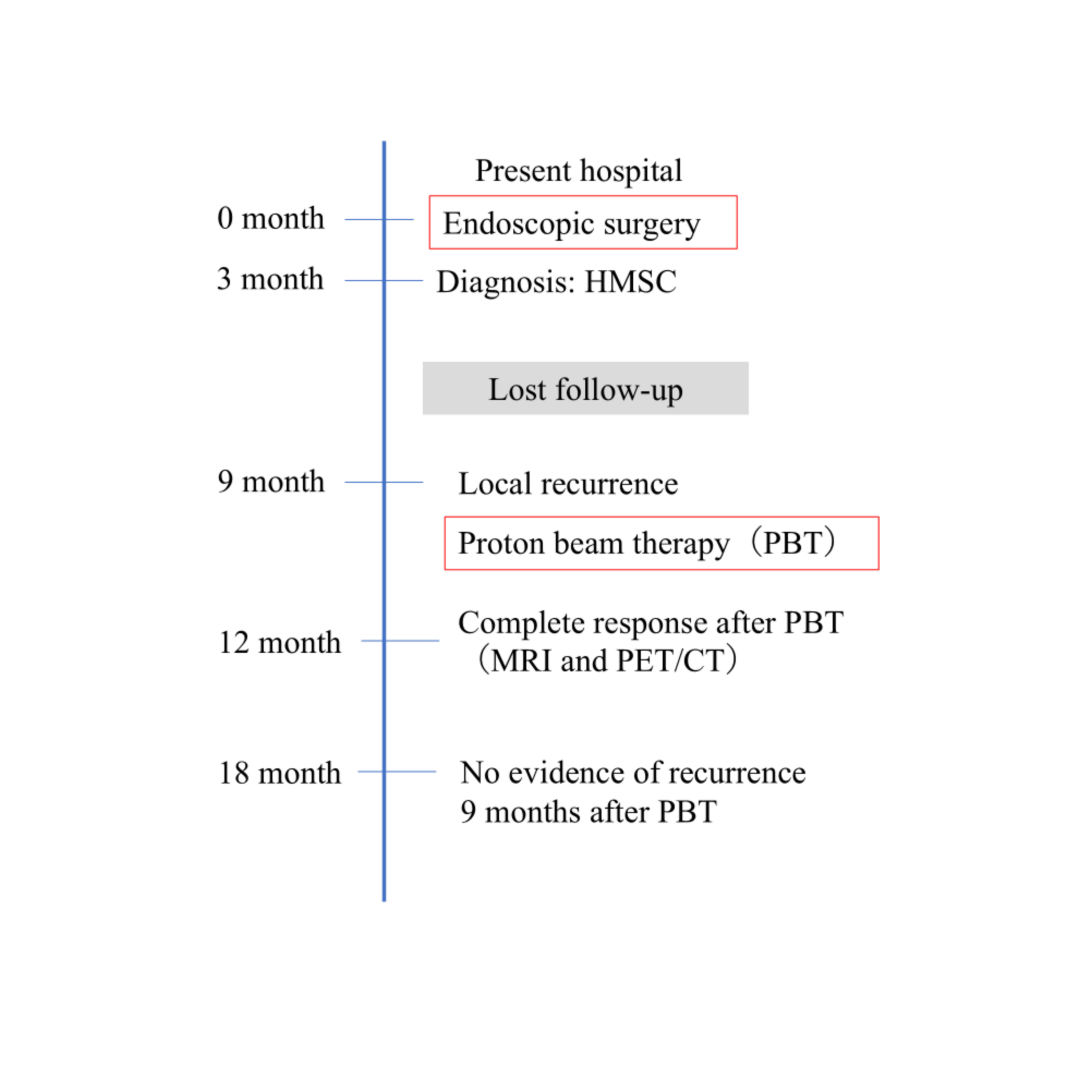
Clinical course of the patient HMSC, human papillomavirus-related multiphenotypic sinonasal carcinoma; MRI, magnetic resonance imaging; PET/CT, positron emission tomography/computed tomography

The key objectives of the patient are presented in a brief timeline table (Figure 6).

## Discussion

Historical and pathological background

Boland et al. detected high-risk HPV in two cases of high-grade adenoid cystic carcinoma. The carcinoma was located in the nasal cavity in both of these HPV-positive cases [12]. Bishop et al. have identified HPV-positive carcinomas with specific pathological features in the sinonasal tract [7]. These distinctive sinonasal tumors were classified as “HPV-related sinonasal carcinomas with adenoid cystic-like features” in the 2017 WHO Classification of Head and Neck Tumors [13]. Later, morphological variations were added, and the name was changed to HMSC [10].

HMSC has the histological features of both adenoid cystic carcinoma-like salivary gland carcinoma and squamous cell carcinoma. The term “multiphenotypic” refers to the multiple cell differentiation lineages often seen in a single tumor. The proliferation of basaloid cells, such as solid sheets, lobules, and cribriform nests, is a conclusive feature of the ACC variant [8]. The salivary gland nature of these tumors is characterized by evidence of both ductal and myoepithelial components of salivary gland tumors, including ACC. Immunohistochemistry tests of the myoepithelial cells are often positive for cytokeratin, S100, actin, calponin, and p63. Squamous dysplasia of the surface epithelium lining the sinonasal tract forms squamous carcinoma. These multiphenotypic tumor cells also consistently exhibit a high mitotic rate and tumor necrosis. Because these features are also observed in ACC, HMSC needs to be distinguished from ACC. MYB or MYBL1 fusion is often detected in ACC, and HMSC does not exhibit these rearrangements [8]. Although p16 immunochemistry is diffusely positive in HMSC, this marker is not specific for diagnosis by itself, as it is also positive in ACC [14]. The presence of high-risk HPV is diagnostic of HMSC, including previously detected types 16, 33, 35, and 56 and mainly HPV type 33 [10]. DNA hybridization, RNA hybridization, and/or PCR-based assays are required to confirm the presence of HPV in sinonasal carcinomas [8]. In this case, we performed the RNA-ISH assay and simultaneously detected high-risk HPV (16, 18, 26, 31, 33, 35, 39, 45, 51, 52, 53, 56, 58, 59, 66, 68, 73, 82). We were able to prove the presence of the HPV gene in tumors, but did not reveal the HPV types.

Clinical behavior and recurrence

HMSC is slightly more common in women [8]. The mean age at diagnosis is approximately 50 years, with a range of 28-90 years [10]. Nasal obstruction and epistaxis are the main symptoms [9]. The original tumor sites are often the nasal, maxillary, and ethmoidal sinuses [9,10]. HMSC is often an early T-stage cancer localized to the sinonasal tract. No metastasis was observed in the HMSC at the time of diagnosis. No deaths due to the tumor have been reported in the literature [10]. Therefore, HMSC is described as having a mild clinical course [9,10]. However, although the clinical course is indolent, HMSC is relatively prone to local recurrence after treatment [9,10]. A previous large case study reported that 14 of 39 (36%) patients observed after the initial treatment developed local recurrence [10]. In previous analyses of selected cases, the local recurrence rate was 36.4%, and there was no lymph node metastasis or minimal distant metastasis [9]. Local recurrence of HMSC often occurs between 24 and 60 months after treatment [9]. However, in this case, the patient was lost to follow-up after surgery, which led to the late detection of early local recurrence. Therefore, it is important to closely monitor the progression of HMSC after primary treatment.

Surgical approaches combined with radiotherapy

To date, most patients diagnosed with HMSC have undergone surgery with or without adjuvant radiotherapy. In a single-center study, 33 of the 38 patients underwent surgery or surgery followed by radiotherapy as the initial treatment [10]. Beaumont et al. described that endoscopic surgery with adjuvant radiotherapy was performed in three cases, and good control was obtained when invasion was suspected before surgery. Therefore, the tumor size and appearance on imaging were not determined by endoscopic dissection [15].

The success of Beaumont et al. with endoscopic resection combined with radiotherapy underscores the possible benefit of adjuvant therapy, which was absent in the present case. Preoperative imaging does reliably predict the depth of infiltration, thus complicating complete surgical margins in endoscopic approaches. In reported cases in which the tumor was limited to the nasal cavity, recurrence after endoscopic resection was observed at another site in the ipsilateral nasal cavity [16,17]. In the present case, the base of the tumor was the nasal septum at the time of the initial surgery; however, a recurrent lesion was found around the ostiomeatal unit. Despite apparent localization, recurrence occurring at a distinct anatomical site highlighted the infiltrative potential of HMSC, even in early-stage disease. Additional radiation therapy should have been performed because sufficient margins were not secured.

Although adjuvant radiotherapy tended to extend the disease-free interval, no association was found between the treatment modality and recurrence rate [9]. In most reported cases, surgery was performed for recurrent HMSC lesions [10]. Miyamaru et al. [16] reported that a recurrent tumor in the inferior turbinate was removed using endoscopic endonasal surgery. Morishita et al. [17] performed partial maxillectomy and reconstruction for recurrent nasal floor and nasal septal disease.

In the present case, skull base surgery requiring enucleation would have been required to treat the recurrent lesion. Surgery was not feasible within sufficient margins, and organ function may not have been preserved given the anatomical complexity.

Novelty and significance of proton beam therapy in this case

Radiotherapy (RT) is used to treat nasal cavity and paranasal sinus malignancies in patients who are not candidates for primary surgery, those with unresectable recurrent lesions [11].

Charged-particle therapy (proton beam therapy or heavier ion therapy) has dosimetric advantages over conventional radiotherapy. Proton beam therapy provides advantages such as rapid dose fall-off at a specific depth beyond the Bragg peak and higher relative biological effectiveness. This increases radiation dose conformity in the primary target tissue and maximally protects adjacent healthy structures [18]. A large-scale study showed that proton beam therapy is a safe and effective treatment for sinonasal tumors [18]. Compared with IMRT, proton beam therapy has been shown to provide improved locoregional control and disease-free survival [11].

Although sinonasal ACC has a poor prognosis because of its complex anatomy and resistance to RT, particle beam therapy (proton beam therapy and carbon-ion therapy) produced satisfactory local control and no acute toxicities of grade 3 or higher [19]. Although the radiation sensitivity of HMSC was unclear, proton beam therapy was performed in accordance with the procedure for ACC, as ACC is known to be highly resistant to radiation therapy. Good local control was achieved, and there were no acute adverse events over grade 3.

This study has some limitations. First, because this was a single-case report, it is unknown whether similar therapeutic effects and mild adverse events of proton beam therapy can be expected in other HMSC cases. Second, this report showed no evidence of recurrence after proton beam therapy; however, the follow-up duration was only nine months. Local recurrences with long disease-free intervals are common in HMSC [8,10]. The therapeutic effects of proton beam therapy cannot be guaranteed to be permanent, and lifelong follow-up is required after treatment. Third, there is limited particle beam therapy equipment, and such advanced technology is not available in many areas. This therapy is associated with high costs for patients and healthcare institutions [20]. These limitations must be considered when applying this method to other HMSC cases.

## Conclusions

This case illustrates the potential of proton beam therapy to achieve durable local control in recurrent HMSC and highlights the importance of integrating multimodal imaging in post-treatment monitoring patients. Despite its generally indolent course, HMSC carries a significant risk of local recurrence, warranting complete surgical resection and long-term surveillance. Proton beam therapy may offer a viable organ-preserving strategy for recurrent HMSC, particularly in anatomically constrained regions where surgery is not feasible.
